# Integration and gene co-expression network analysis of scRNA-seq transcriptomes reveal heterogeneity and key functional genes in human spermatogenesis

**DOI:** 10.1038/s41598-021-98267-3

**Published:** 2021-09-27

**Authors:** Najmeh Salehi, Mohammad Hossein Karimi-Jafari, Mehdi Totonchi, Amir Amiri-Yekta

**Affiliations:** 1 Department of Genetics, Reproductive Biomedicine Research Center, Royan Institute for Reproductive Biomedicine, ACECR, Tehran, Iran; 2 School of Biological Science, Institute for Research in Fundamental Sciences (IPM), Tehran, Iran; 3 Department of Bioinformatics, Institute of Biochemistry and Biophysics, University of Tehran, Tehran, Iran

**Keywords:** Computational biology and bioinformatics, Genetics

## Abstract

Spermatogenesis is a complex process of cellular division and differentiation that begins with spermatogonia stem cells and leads to functional spermatozoa production. However, many of the molecular mechanisms underlying this process remain unclear. Single-cell RNA sequencing (scRNA-seq) is used to sequence the entire transcriptome at the single-cell level to assess cell-to-cell variability. In this study, more than 33,000 testicular cells from different scRNA-seq datasets with normal spermatogenesis were integrated to identify single-cell heterogeneity on a more comprehensive scale. Clustering, cell type assignments, differential expressed genes and pseudotime analysis characterized 5 spermatogonia, 4 spermatocyte, and 4 spermatid cell types during the spermatogenesis process. The UTF1 and ID4 genes were introduced as the most specific markers that can differentiate two undifferentiated spermatogonia stem cell sub-cellules. The C7orf61 and TNP can differentiate two round spermatid sub-cellules. The topological analysis of the weighted gene co-expression network along with the integrated scRNA-seq data revealed some bridge genes between spermatogenesis’s main stages such as DNAJC5B, C1orf194, HSP90AB1, BST2, EEF1A1, CRISP2, PTMS, NFKBIA, CDKN3, and HLA-DRA. The importance of these key genes is confirmed by their role in male infertility in previous studies. It can be stated that, this integrated scRNA-seq of spermatogenic cells offers novel insights into cell-to-cell heterogeneity and suggests a list of key players with a pivotal role in male infertility from the fertile spermatogenesis datasets. These key functional genes can be introduced as candidates for filtering and prioritizing genotype-to-phenotype association in male infertility.

## Introduction

Spermatogenesis is a highly organized and complex process of differentiation events that produces sperm from the primordial germ cells^1^. Sperm production occurs in the seminiferous tubules, is a continuous process that begins at puberty and continues throughout life^2^. This productivity depends on the activity of the spermatogonia stem cells (SSC), which are the stem cells of adult testicular tissue^3^. The SSCs are capable of perpetual self-renewal and differentiation division, which preserves the stem cell pool and spermatogenesis fuel, respectively^3,4^. Then, differentiating spermatogonia cells divide mitotically and produce two diploid spermatocytes, followed by two meiosis and the spermiogenesis process to produce haploid spermatids and sperm, respectively^1,4^. Between 1500 and 2000 genes are thought to play a role in controlling spermatogenesis and genetic changes in these genes are expected to impair male fertility^5,6^. Currently, the genetic diagnosis for male infertility includes screening a short list of candidate genes that should be expanded^7–9^. Hence, a high-resolution profile of gene expression signatures in the process of spermatogenesis can be a starting point for solving male infertility^10^.

Gene expression profiling assays, such as typical microarray or RNA-sequencing (RNA-seq) have been widely used to investigate the changes in testicular gene expression from birth to adulthood^11–14^, and in the molecular mechanisms involved in male infertility^15,16^. These studies rely on the bulk RNA analysis of mixed aggregates of spermatogenic cells, that provide the average expression signal for a pool of different cell types^17,18^. Therefore, they lose within and between cell type diversity or rare cell phenotypes^17^. To isolate spermatogenic cell types, some common approaches such as fluorescence-activated cell sorting (FACS), magnetic activated cell sorting (MACS), and STA-PUT are used^17,19^. However, these methods can only separate some types of spermatogenic cells and cannot isolate high-purity homogeneous spermatogenic cells from all types^10,20^.

Single-cell RNA sequencing (scRNA-seq) provides the transcriptome profiles of individual cells that can investigate the variation within and between cell types and reveal rare cell types^17^. In the last few years, some studies have examined the transcriptome profiles of different cell types in human testicular tissue using scRNA-seq. Most of these studies have investigated spermatogenesis single-cell transcriptome in only fertile individuals or obstructive azoospermia (OA) patients^21–28^. A few number of studies in non-obstructive azoospermia (NOA) patients have been reported^29,30^. FACS, MACS, and STA-PUT were used to sort individual cell types before scRNA-seq in some studies^21–23,29^. However, scRNA-seq can examine thousands of individual cells in the steady-state of spermatogenesis without the need for prior sorting^22,23,25–27,29^. Also, single-cell transcriptomes of infants, juvenile and adult males were profiled to investigate the changes in the spermatogenesis cell types at birth, during puberty, and adulthood^23,25,27^. The common idea in all of these studies was to identify cell types based on the key markers expressions, find differentially expressed genes (DEGs) in each cell type, and enrich their biological functions which showed significant heterogeneity within and between spermatogenesis cell types.

In this study, we integrated the scRNA-seq data of human spermatogonia, spermatocyte, spermatid sorted cells^22^, and steady-state spermatogenic cells^22,29^. The integrated analysis of these datasets provides a more comprehensive profile of spermatogenesis prossess^31^. Then clustering, cell type assignments, DEGs, enrichment, and pseudotime trajectory analysis were performed to characterize cell heterogeneity. Furthermore, a related gene co-expression network was generated, and its topological analysis revealed bridge genes in this process. The role of these bridge genes in male infertility makes them candidates for filtering and prioritizing genotype-to-phenotype association and gene expression alterations in male infertility.

## Results

### Clustering of integrated spermatogenesis dataset

**Figure 1:**
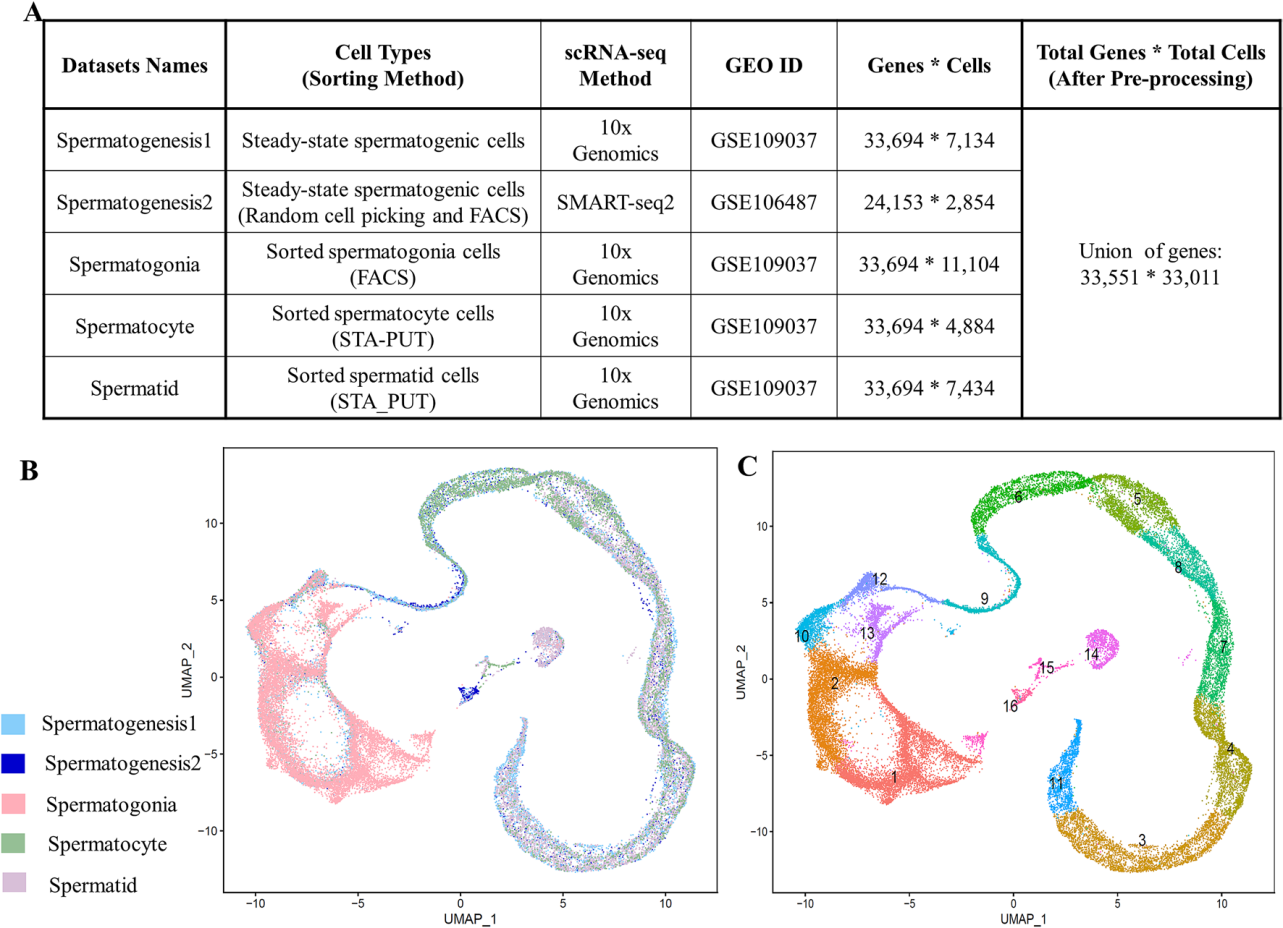
Profiling and integrating testicular datasets. (**A**) Datasets information of adult human testicular cells that were integrated and analyzed, such as sorting methods, scRNA-seq methods, GEO ID, and the number of genes and cells for each data set were listed. (**B**,**C**) UMAP plot of integrated human testicular cells. Cells are colored based on (**B**) the original datasets, (**C**) clustering results.

The diverse human spermatogenesis scRNA-seq datasets, including spermatogonia, spermatocyte, spermatid sorted cells, and steady-state spermatogenic cells were collected from the GEO database. The cell types, sorting methods, scRNA-seq methods, GEO ID, and the initial number of genes and cells in each dataset were summarized in Fig. 1A. After pre-processing, 33,011 spermatogenic cells were gathered. The integrated datasets in the UMAP low dimensional space showed that similar cells in different datasets were placed together in the UMAP space (Fig. 1B). Each dataset in the UMAP space of integrated data was presented in detail in Figure S1. The Spermatogenesis1 dataset which belongs to steady-state spermatogenic cells^22^, depicted the greatest similarity with the integrated data in the UMAP space (Fig. 1B, Fig. S1A). On the other hand, some of the Spermatocyte and Spermatid dataset cells, that were isolated using the STA-PUT method, are mixed in the UMAP space of integrated data (Fig. 1B, Fig. S1D,E). The unsupervised, graph-based clustering revealed 16 clusters of testicular cells in this integrated data which is shown in the UMAP plot (Fig. 1C).

### Cell type assignment shows heterogeneity among testicular cells

**Figure 2:**
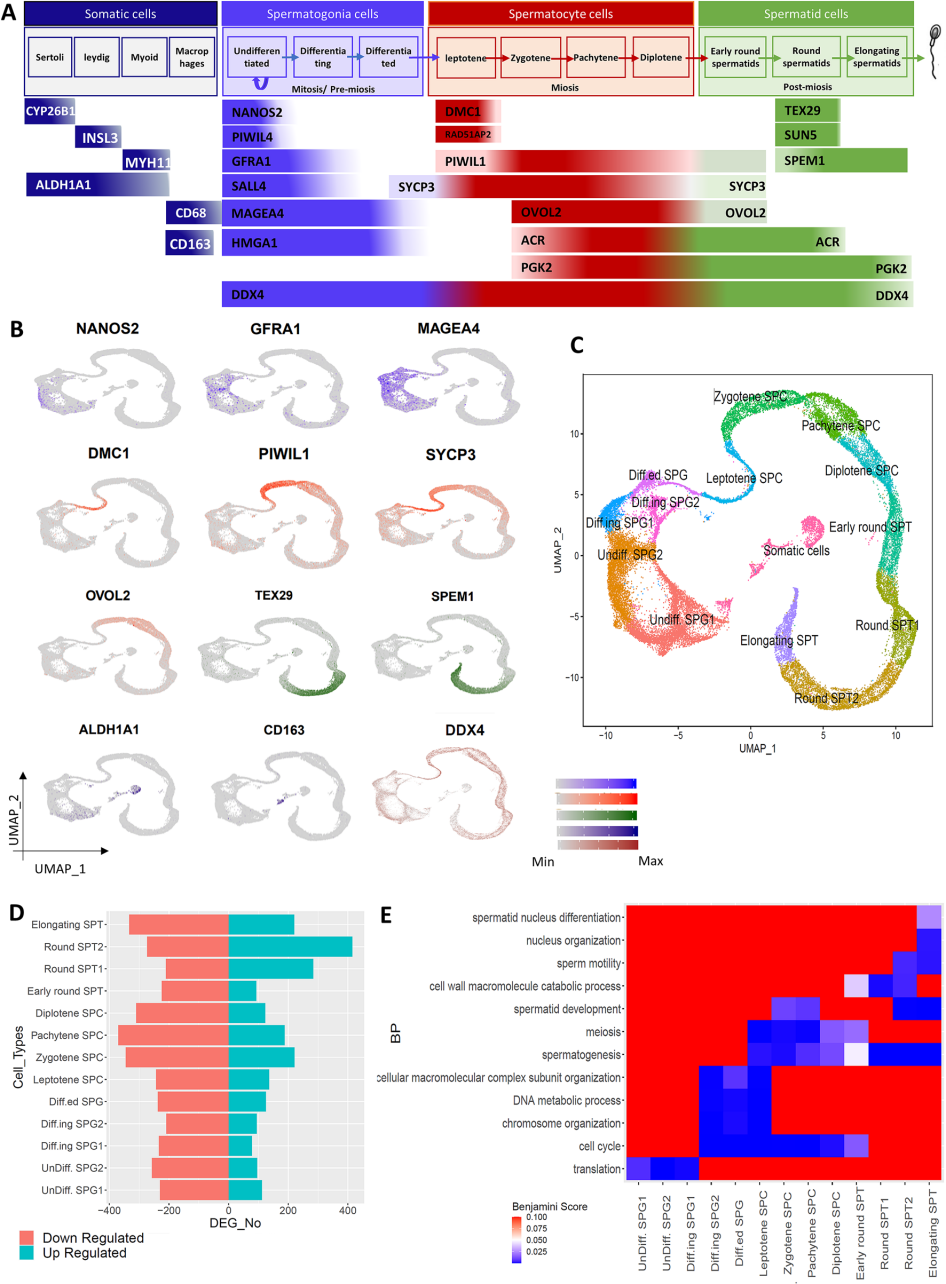
Cell type assignment of clusters. (**A**) Gene markers of testicular cells were categorized based on different somatic, spermatogonia, spermatocyte, and spermatid cells, (**B**) gene expression patterns of these markers on the UMAP space which were colored based on the A part categorization, (**C**) cell type assignment of clusters based on gene markers expression patterns, (**D**) the number of up- and down-regulated genes in different germ cell types, (**E**) the biological processes enrichment for up-regulated genes of different germ cell type clusters.

Due to the importance of cell-type assignment to the clusters, the expression of some known markers of testicular germ and somatic cells were evaluated (Fig. 2A). The NANOS2 and PIWIL4 are major genes for SSC maintenance and are expressed in self-renewing SSC^25,29,32–34^. These marker genes were specifically expressed in cluster-1 and -2 which were named Undiff. SPG1 and Undiff. SPG2, respectively (Fig. 2B,C, Fig. S2). GFRA1 and SALL4 are well-known markers for both undifferentiated and differentiating SSCs^35^ which were expressed in cluster-1, -2, -10, and -13. So, cluster-10 and -13 were assigned to differentiating cells and termed as Diff.ing SPG1 and Diff.ing SPG2, respectively (Fig. 2B,C, Fig. S2). Cluster-12 was identified as a differentiated spermatogonia cell cluster (Diff.ed SPG) due to the MAGEA4 and HMGA1 expression in cluster-1, -2, -10, -12, and -13 for all spermatogonia cells (Fig. 2B,C, Fig. S2)^29,35,36^. DMC1 and RAD51AP2 are mitotic genes expressed at the leptotene stage^37^. Accordingly, cluster-9 with the highest expression level of these genes belonged to leptotene cells, denoted as the Leptotene SPC cluster (Fig. 2B,C, Fig. S3). PIWIL1 expression is initiated from spermatocyte to spermatid cells with the highest expression level in zygotene and pachytene^38^. Also, SYCP3 was upregulated from differentiated spermatogonia cells to the early round spermatid stage^39^. OVOL2 is expressed from zygotene to diplotene, relating to the presence of the sex body during mammalian male meiosis^40^. Accordingly, cluster-6, -5, -8, and -7 were recognized as the zygotene, pachytene, diplotene stages of spermatocytes and the early round spermatids, respectively, that were named as Zygotene SPC, Pachytene SPC, Diplotene SPC and Early round SPT (Fig. 2B,C, Figs. S3, S4). TEX29 and SUN5 genes can be observed in the round spermatids^29^, which were expressed in cluster-3 and -4 (denoted as Round SPT1 and Round SPT2). Furthermore, ACR and PGK2 presented in zygotene to round spermatids and elongating spermatids, respectively^22,41,42^. SPEM1 is expressed in the late stages of spermatid^43^. Thus, cluster-11 corresponded to the last stage of spermatid, which was named as Elongating SPT (Fig. 2B,C, Fig. S4). To detect somatic cells clusters, the expression pattern of CYP26B1 as Sertoli^44^, INSL3 as Leydig^45^, MYH11 as myoid^46^, and ALDH1A1 as Sertoli, Leydig and myoid markers^47,48^, were evaluated. Also, CD68 and CD163 are known markers of macrophages. These investigations showed cluster-13, -14, -15 as somatic cell clusters. On the other hand, the DDX4 gene expression pattern, as germ cells marker, confirmed the somatic cell clusters assignment. All of these cell clustering analyses on datasets and cell-type assignments are summarized in Table 1.

**Table 1:**
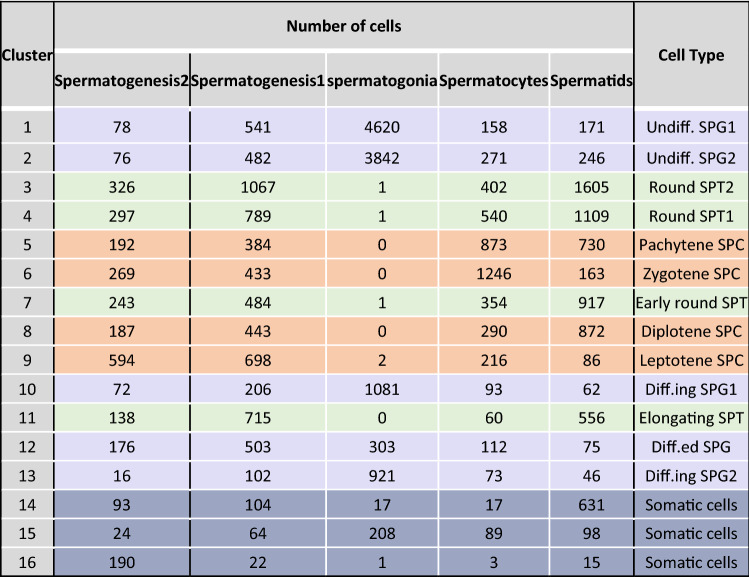
Characteristics of clusters. The numbers of cells for each dataset, cluster, and cell type assignment for each cluster were specified.

The rows are colored based on the cell types.

The expression patterns of DEGs were compared among all cell-type clusters (Table S1). The number of up and down-regulated genes (or positive and negative DEGs) in all germ cell types (13 clusters) were measured and compared with each other. Among all spermatogenic cell clusters, Round SPT2 and Round SPT1 displayed the most up-regulated genes with 415 and 284 genes, respectively (Fig. 2D). On the other hand, Pachytene SPC and Zygotene SPC presented the most down-regulated genes with 370 and 345 genes (Fig. 2E).

The cell assignment results demonstrated five spermatogonia cells. Among them, the Undiff. SPG1, Undiff. SPG2 and Diff.ing SPG1 positive DEGs were enriched especially for biological processes (BPs) related to translation (Fig. 2E). Translation in undifferentiated stem cells is usually kept low and must be strictly regulated^49^. Nevertheless, stem cells need to maintain the proper expression level of the main stem factors to keep their specific properties and characteristics^49^. Also, a higher RNA production in mouse spermatogonia cells was reported in earlier studies^50^. The Diff.ing SPG2 and Diff.ed SPG cells were enriched with terms of the cell cycle, chromosome organization, DNA metabolic process, and cellular macromolecular complex subunit organization (Fig. 2E). The cell cycle or cell-division cycle is started in differentiating spermatogonia cells with mitotic division and continued in spermatocyte cells with meiosis division^51^. During mitosis, extensive chromosome organization is needed to transport genetic material to the daughter cells^52^. In the Leptotene SPC cells, BPs of spermatogenesis and meiosis were enriched in addition to Diff.ed SPG BPs (Fig. 2E). The meiosis process was the main BP in the spermatocyte cells. The cell wall macromolecule catabolic process genes were highly expressed in Round SPT1 and Round SPT2 (Fig. 2E). Furthermore, spermatid development and sperm motility were up-regulated in Round SPT2 and Elongating SPT. Finally, in Elongating SPT cells, spermatogenesis, spermatid development, sperm motility, nucleus organization, and spermatid nucleus differentiation were enriched (Fig. 2E). The BP enrichment seems reasonable since the closer cells in the differentiation process, the more similar BPs are enriched.

### Developmental ordering of spermatogenesis cells

**Figure 3:**
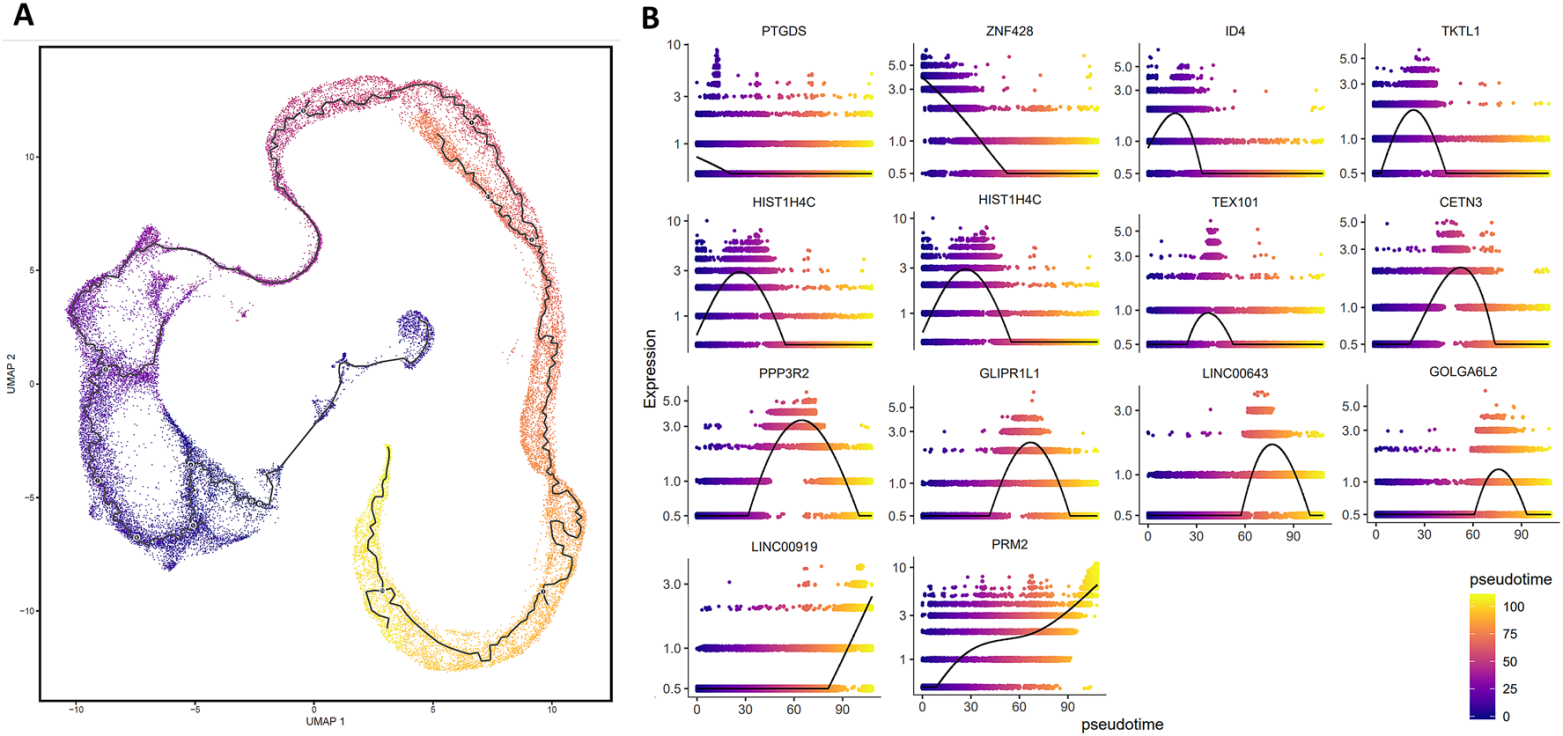
Developmental ordering of spermatogenesis cells. (**A**) The pseudotime analysis of testicular cells on the UMAP space, purple cells represent the beginning of the path and yellow cells represent the end of the path (**B**) the expression of top positive DEGs in each cluster along the pseudotime.

The developmental order of these cells and clusters on the UMAP space were in agreement with spermatogenesis cell order (Fig. 3A). The PTGDS and ZNF428 were the top up-regulated genes in somatic and Undiff. SPG1, which were expressed at the same time (Fig. 3B). Then ID4, TKTL1, HIST1H4C, HIST1H4C, TEX101 CETN3, PPP3R2, GLIPR1L1, LINC00643, LINC00919, GOLGA6L2, PRM2 were expressed sequentially which were the top up-regulated genes in Undiff. SPG2, Diff.ing SPG1, Diff.ing SPG2, Diff.ed SPG, Leptotene SPC, Zygotene SPC, Pachytene SPC, Diplotene SPC, Early round SPT, Round SPT1, Round SPT2, and Elongating SPT cell clusters, respectively (Fig. 3B).

### Weighted gene co-expression network indicates bridge genes between testicular cells

**Figure 4:**
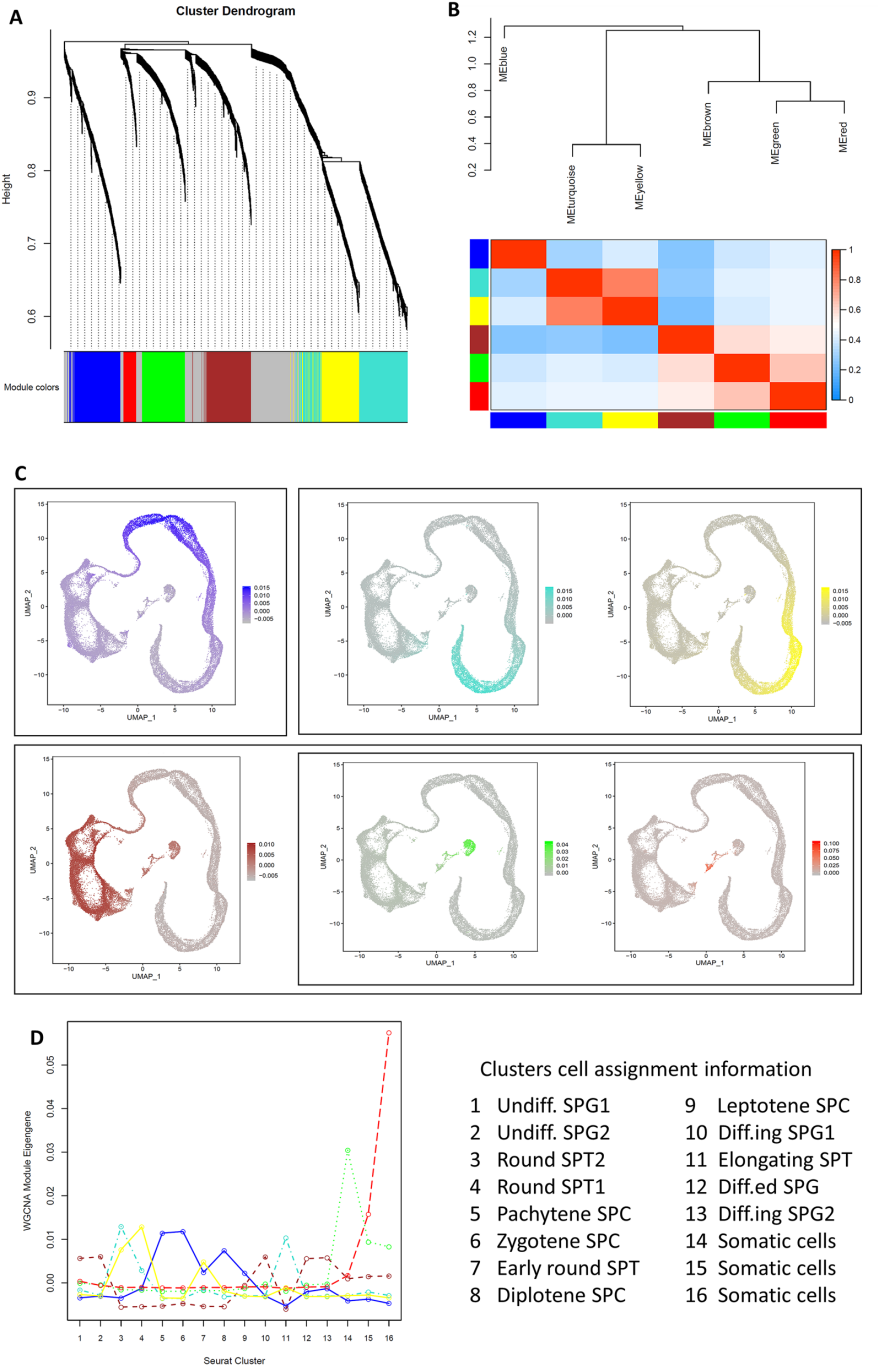
Weighted gene co-expression network analysis. (**A**) The clustering dendrogram of the weighted gene co-expression network. The resulted modules are depicted in different colors of blue, turquoise, yellow, brown, green, red, and gray. The gray modules gene lacked similar co-expression to other genes which were removed from more analysis. (**B**) the eigengene dendrogram and eigengene adjacency heatmap of modules, (**C**) the gene expression patterns on the UMAP space for each module with their corresponding colors, (**D**) the eigengene of each module in each cluster.

The clustering dendrogram of genes in the weighted gene co-expression network (WGCN) resulted in 6 modules (Fig. 4A). The eigengene dendrogram and eigengene adjacency heatmap displayed the inter-modular relationships which revealed a high correlation between turquoise and yellow modules (Fig. 4B). Also, there was a correlation between the red and the green modules and between these modules with the brown one. The brown module eigengenes location on the UMAP space and its higher values in cluster-1, -2, -10, -12, and -13 indicated that this module related to the co-expressed genes in spermatogonia cells (Fig. 4C,D). The blue module eigengenes fitted to the location of the spermatocyte cells on the UMAP and cluster-5, -6, -8, and -9 (Fig. 4C,D). These results for turquoise and yellow modules displayed that these modules were related to co-expressed genes in spermatid cells. The co-expressed genes in the somatic cells were presented in red and green modules which revealed higher expression in cluster-14, -15, and -16 (Fig. 4C,D).

**Figure 5:**
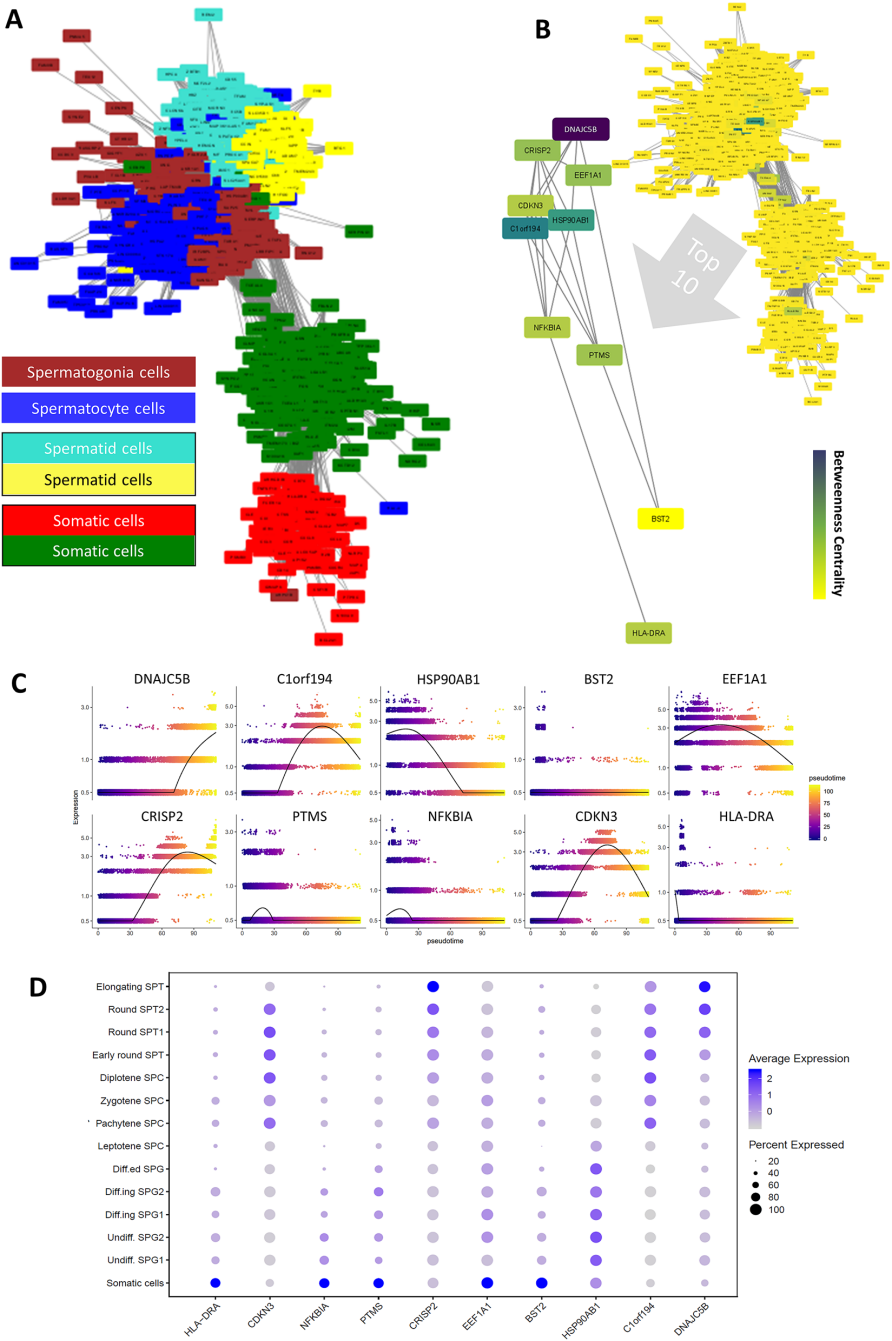
Betweenness centrality analysis of the weighted gene co-expression network. (**A**) The presentation of the weighted gene co-expression network. The relation between colored modules and cell types were shown in the inset figure. (**B**) The co-expression network was colored based on the betweenness centralities from yellow to purple. The top ten genes with the highest betweenness centralities are highlighted. (**C**,**D**) The expressions of these top betweenness centrality genes along (**C**) the pseudotime and (**D**) the cell-types clusters.

The WGCN of the integrated data was constructed and shown with Cytoscape (Fig. 5A). The gene co-expression network is colored based on the betweenness centrality (BC) value for each node (Table S2) and its top ten nodes, DNAJC5B, C1orf194, HSP90AB1, BST2, EEF1A1, CRISP2, PTMS, NFKBIA, CDKN3, and HLA-DRA with the highest *p*-values were highlighted (Fig. 5B). The results demonstrated all these genes were expressed in all cell-type clusters with different levels. BST2, EEF1A1, PTMS, NFKBIA, and HLA-DRA revealed higher expression at the beginning of the pseudotime trajectory in somatic cells (Fig. 5C,D). HSP90AB1 was one other bridge gene in this network that was particularly expressed in spermatogonia cells. C1orf194 and CDKN3 were specially expressed in the middle of the pseudotime trajectory and spermatocyte cell clusters (Fig. 5C,D). DNAJC5B (with the highest BC value) and CRISP2 were other bridge genes that were expressed in the spermatid cell clusters especially the Elongating SPT cluster (Fig. 5C,D). Then these analyses were performed between brown and blue modules in the WGCN to find bridge genes between the spermatogonia and spermatocyte cells as sequential cell types in spermatogenesis. The mentioned BCs and *p*-values were presented in Table S3. C1orf194, HSP90AB1, MFSD6L, TPD52L3, PTMA, PHF7, BOLL, TEX40, C6orf48, and NDUFAF3 were detected as the bridge genes between the brown and blue modules in the WGCN (Fig. S6A,B). The gene expression along the time trajectory and clusters (Fig. S6C,D) showed most of these genes expressed in the middle of time and spermatocyte cells. Then, the bridge genes between spermatocyte and spermatid cells were evaluated, using BC between related modules (Fig. S7A, Table S4). The centrality analysis identified DNAJC5B, C1orf194, CDKN3, CRISP2, MFSD6L, CCDC89, CALM2, TPD52L3, SPACA7, and RCN2 as bridge genes (Fig. S7B). These genes expressions were well-distributed between both cell type clusters and along the pseudotime trajectory (Fig. S7C,D).

## Discussion

In this study, we integrated diverse scRNA-seq datasets of more than 33,000 testicular cells, to identify pure and comprehensive cell profiles for spermatogenesis. Some of these datasets were retrieved from Hermann et al.study^22^, which contains the steady-state of spermatogenesis and three sorted spermatogenic cell types that are not integrated. One other steady-state dataset was retrieved from Wang et al*.* study^29^. The value of integrating and re-analyzing these datasets is due to genetic diversity and different developmental timing between different individuals. Furthermore, in each study, only a few samples were evaluated that tissues were available due to a disease or trauma other than infertility^10^. Our data integration led to the coverage of similar cell types in different datasets. However, sorted spermatocyte and spermatid data overlapped which can be a drawback of the STA-PUT method to isolate pure cells^10,53^. The integration, in our study, led to 16 clusters within the spermatogenesis complex process. The goal of scRNA-seq datasets integration is to improve cell classifications and identify differences in cell type dependent gene expression^31^.

The evaluation of marker gene expression identified two, two, and one clusters for undifferentiating, differentiating, and differentiated spermatogonia cells, respectively. While, the number of spermatogonia clusters in the Spermatogenesis1^22^ and Spermatogenesis2^29^ datasets were four and three, respectively^22,29^. The spermatogonia cells presented fewer up-regulated genes than down-regulated ones that were engaged in the translational process and started the cell cycle. The UTF1 and ID4 genes are known marker genes for SSC^54,55^ that were differentially expressed in Undiff. SPG1 and Undiff. SPG2 clusters, respectively. A similar result showed these genes marked distinctly with a partial overlap in the undifferentiated spermatogonia cells, which proved the heterogeneity in these cells^55^. ASB9 gene was detected as a top DEG in Diff.ing SPG1 cluster which is consistent with its expression in early differentiating spermatogonia cells^25^. Diff.ing SPG2 belongs to the late differentiating spermatogonia cells, due to the similarities in top DEGs with Diff.ed SPG cells. All results insist on heterogeneity within the spermatogonia cell population which was declared in some previous studies^56–59^. The four cell types of spermatocytes (leptotene, zygotene, pachytene, and diplotene) were identified distinctly which their DEGs significantly enrich meiosis BP. These different stages of meiotic prophase I associated with genes down-regulation that is consistent with low RNA production during early meiosis in humans^60^ and mice^50^. Whereas the Spermatogenesis1^22^ and Spermatogenesis2^29^ datasets alone revealed four and seven spermatocyte clusters, respectively^22,29^. The seven spermatocyte clusters in Spermatogenesis2 were divided into three leptotene, one zygotene, one pachytene, one diplotene, and one mixture of spermatocyte cell clusters^29^. Four spermatid clusters demonstrated the heterogeneity in spermatid cells with one cluster for early-round, two for round, and one for elongating spermatid cells. In addition to spermatocytes, the Early round SPT cluster also enriched meiosis BP which produces round spermatids^61^. The C7orf61 and TNP1 are two known round spermatids markers that belonged to top DEGs of Round SPT1 and Round SPT2, respectively. These results indicate the presence of heterogeneous spermatid cells during the spermatogenesis process which presented many up- and down-regulated genes compared to other spermatogenesis cells. On the other hand, the Spermatogenesis1^22^ and Spermatogenesis2^29^ datasets presented seven and four spermatid cell clusters, respectively^22,29^. The expression of the top DEG of each cluster in pseudotime proved another confirmation on the cell type assignment and ordering. Based on these results the clustering of the integrated scRNA-seq of the spermatogenic cells led to more comprehensive clustering than each of those datasets separately.

The "Guilt by Association" is one of the concepts that provide the use of gene co-expression networks to identify gene functions and molecular mechanisms in biological processes^62^. Gene co-expression network on scRNA-seq data can find functional modules related to a specific state^63,64^. In this regard, the WGCN analysis detected six modules. Adaptation of these six modules expression patterns with cell clusters and eigengene dendrograms led to the attribution of these co-expressed gene modules to the main stages of testicular cells, including somatic, spermatogonia, spermatocyte, and spermatid cells. Topological analysis of a cell-type-specific gene co-expression network can be useful to find the main functional genes between modules^63^. Among the network topological analysis, BC represents the influence of a node on its neighbors and the spread of information, in other words, a node with a high value of BC can be the bridge point between network modules^65,66^. The BC investigation of WGCN of these integrated testicular scRNA-seq datasets showed DNAJC5B, C1orf194, HSP90AB1, BST2, EEF1A1, CRISP2, PTMS, NFKBIA, CDKN3, and HLA-DRA were the top ten genes with the highest BC and *p*-values. Interestingly, studies have shown that most of these genes have played a role in infertility disorders. C1orf194 was differentially expressed in the asthenozoospermic infertile group in comparison to the normozoospermic infertile group^67^. HSP90AB1 interacted with the catalytic domain of Kdm3a, that mutant Kdm3a can cause male infertility in mice^68^. Furthermore, the Hsp90ab1 gene lacking was reported to cause embryo death during implantation in mice^69^. The EEF1A1 heterozygous mutation led to spermatogenesis arrest phenotype and male infertility in tilapia^70^. The low CRISP2 expressions in asthenozoospermic^71,72^ and teratoasthenozoospermic^73^ patients were reported. An association was identified between NFKBIA gene polymorphisms and idiopathic male infertility risk^74^. The expression of the CDKN3 gene was reduced in teratozoospermic men^75^. GWAS studies showed HLA-DRA gene-related SNPs were significantly related to Nonobstructive Azoospermia^76,77^. Interestingly, five of these genes are highly expressed in the somatic cells which is consistent with the high effects of somatic cells on the different stages of spermatogenesis^78^. Then to find specific bridge genes between the main stages of spermatogenesis, we zoomed in sequential stages of testicular cell genes in the WGCN. The top ten BC genes between spermatogonia and spermatocyte modules were C1orf194, HSP90AB1, MFSD6L, TPD52L3, PTMA, PHF7, BOLL, TEX40, C6orf48, and NDUFAF3. The top two BC genes between these modules, C1orf194, and HSP90AB1, were similar to the top BC genes of the global WGCN. A down-regulation of TPD52L3 was reported in oligozoospermia^79^. Disruption of Phf7 caused infertility in male mice by decreasing sperm count and increasing abnormal sperm ratio^80^. The relation of BOLL deletion or mutation with unfunctional sperm production that led to infertility has been reported in different studies^81–84^. The expression of TEX40, a calcium entry protein, is reduced in asthenozoospermic males^85^ and targeted disruption of TEX40 led to severe male subfertility in mice^86^. In the next step, the top ten BC genes between spermatocyte and spermatid modules were examined as two sequential modules to find the bridge genes between them. The DNAJC5B, C1orf194, CDKN3, CRISP2, MFSD6L, CCDC89, CALM2, TPD52L3, SPACA7, and RCN2 genes were identified as the top ten BC genes. The four (DNAJC5B, C1orf194, CDKN3, and CRISP2) and three (C1orf194, MFSD6L and TPD52L3) genes between these modules were similar to the top BC genes of the global, and spermatogonial-spermatocyte part of the WGCN, respectively. C1orf194 was detected as the top BC gene in all global, spermatogonial-spermatocyte, and spermatocyte-spermatid parts of the WGCN.

In summary, different testicular scRNA-seq datasets were integrated to construct comprehensive spermatogenesis transcriptome-wide data. The clustering, cell type assignments, DEGs, and pseudotime analysis revealed heterogeneity in spermatogenesis's main stages. Then, the WGCN along with the integrated scRNA-seq data identified functional modules associated with the main stages of spermatogenesis. The BC analysis on this cell-type-specific WGCN discovered some bridge genes between the spermatogenesis main stages such as DNAJC5B, C1orf194, HSP90AB1, BST2, EEF1A1, CRISP2, PTMS, NFKBIA, CDKN3, and HLA-DRA. Some of these bridge genes are highly expressed in the somatic cells, emphasizing the role of somatic cells in spermatogenesis. Available studies about these genes showed that perturbation of these genes led to male infertility disorders, which confirms the functional role of top betweenness genes in this cell-type-specific WGCN. These functional bridge genes can be suggested as candidates for filtering and prioritizing genetic variants and gene expression alterations with the goal of introducing a male infertility panel. So, our study not only offers knowledge about cell-to-cell heterogeneity in spermatogenesis but also introduces key genes between the functional modules of normal spermatogenesis that may play important roles in male infertility disorders. These results can be a starting point for experimental research to investigate the function of these genes in male infertility.

## Methods

### The scRNA-seq datasets and preprocessing

The scRNA-seq datasets related to human spermatogonia, spermatocyte, spermatid sorted cells (GEO: GSE109037)^22^ and steady-state spermatogenic cells (GEO: GSE109037 and GSE106487)^22,29^ with normal spermatogenesis were retrieved from the gene expression omnibus (GEO) repository^87^. The FACS and STA-PUT were used to sort spermatogonia, spermatocyte, and spermatid cells in the library of GSE109037^22^. They extract more than 33,000 sorted and unselected steady-state spermatogenic cells from thirty individuals with normal spermatogenesis and used 10 × Genomics Chromium to perform scRNA-seq (Fig. 1A)^22^. In the study of GSE106487, 2854 testicular cells from nine donors with normal spermatogenesis were analyzed with SMART-seq2 protocol^29^. They used random- and FACS-based cell picking to explore all the cell types in the adult human testis (Fig. 1A)^29^. The Seurat3.2 R package^88^ was used for data analysis. To filter out low-quality cells, at first, cells with less than 200 expressed genes and genes expressed in less than 3 cells were removed. Then, cells with a very low or high number of genes and cells with a high percentage of mitochondrial genes were filtered. Standard preprocessing, normalizing, and identifying 2000 highly variable features were performed individually for each dataset. Finally, 33,011 cells were collected for integration.

### Data integration and analysis

Anchor strategy^89^ was used to integrate these datasets, which were produced across multiple technologies. Finding an accurate set of anchors is the basis for subsequent integration analyses. Thus, these datasets were integrated with 2000 anchors, resulting in a batch corrected expression matrix for all cells. The new integrated matrix was used for scaling and the principal component analysis (PCA). The first 35 principal components (PCs) were selected based on the variance percentage of each PC to perform UMAP non-linear dimension reduction^90^ to visualize, explore and separate cells. The graph-based clustering approach of the Seurat3.2 R package was used to find clusters with a dimensionality of 35 and a resolution of 0.2. The cell type of each cluster was assigned based on the expression of specific markers of spermatogenic cells obtained from the literature.

### Differentially expressed genes and enrichment analysis

To find differentially expressed genes (DEG), the non-parametric Wilcoxon rank sum test^91^ was used. The minimum percentage in both cell groups (min.pct) and the log fold-change of the average expression between the two cell groups (logfc.threshold) were set to 0.25 and 0.5, respectively. The up and down-regulated genes in each cluster in comparison to all other clusters were quantified based on positive and negative averaged log fold-change values, respectively. Up-regulated genes with averaged log fold-change higher than 0.7 and adjusted *p*-value (based on Bonferroni correction) less than 0.05 were selected for enrichment analysis. The Database for Annotation, Visualization, and Integrated Discovery (DAVID) v6.7^92^ was used for gene enrichment analysis. The biological processes (BPs) terms with the lowest Benjamini correction score (adjusted *p*-value) were used to plot the heat map.

### Pseudotime analysis

For pseudotime analysis, the Monocle3 R package was used^93^. The integrated data, dimension reduction, and clustering information were imported from Seurat to the Monocle3 package. To order the cells in pseudotime, Monocle3 learns a trajectory that reconstructs the progress of a cell in a cell differentiation process. After the graph learning, the cells were ordered according to their progress.

### Co-expression network construction and analysis

To reveal correlations between gene expression of these integrated cells, a weighted gene co-expression network (WGCN) was created by the WGCNA R package^94^. To construct the WGCN with scale-free topology, different values of soft thresholding power *β* were assessed for the network topology analysis, and the value of 6 was selected. The Pearson correlation coefficient and the signed network options were used to measure the correlation between the expression of each pair of genes and to maintain only positive correlations, respectively. The topological overlap measure (TOM), which investigates the similarities between gene pairs based on the number of shared neighbors in the resulting co-expression network, was used to identify modules. Modules in the WGCN were depicted in different colors. Genes that lacked similar co-expression to other genes in the network, were assigned to the gray module. So, the gray module was removed from more analysis. The relationships between the detected modules were depicted by module eigengenes that are the first principal component of the expressions in modules. Constructed WGCN was exported to Cytoscape^95^. To find essential genes in this network, the betweenness centrality (BC) of each node was measured. A node with the highest BC value indicates the bridge node in that network^66^. To measure the *p*-value for each gene, the random gene label permuting was used for 100,000 steps. Cytoscape and its plugin CytoNCA^65^, were used for network visualization and centralities measurements, respectively.

Codes are available at https://github.com/nasalehi/scRNAseq_spermatogenesis.

## Supplementary Information

Supplementary Information 1.

Supplementary Information 2.

Supplementary Information 3.

Supplementary Information 4.

Supplementary Information 5.

## Abbreviations

BC: Betweenness centrality
BP: Biological process
DAVID: Database for annotation, visualization, and integrated discovery
DEG: Differentially expressed gene
Diff.ed SPG: Differentiated spermatogonia cell
Diff.ing SPG: Differentiating spermatogonia cell
FACS: Fluorescence activated cell sorting
GEO: Gene expression omnibus
MACS: Magnetic activated cell sorting
NOA: Non-obstructive azoospermia
OA: Obstructive azoospermia
PC: Principal component
PCA: Principal component analysis
RNA-seq: RNA-sequencing
scRNA-seq: Single-cell RNA sequencing
SPC: Spermatocyte
SPT: Spermatid
SSC: Spermatogonia stem cells
TOM: Topological overlap measure
Undiff. SPG: Undifferentiated spermatogonia cell
WGCN: Weighted gene co-expression network
