# Supplementary material for: Integration and gene co-expression network analysis of scRNA-seq transcriptomes reveal heterogeneity and key functional genes in human spermatogenesis
Source: Sci Rep. 2021 Sep 27;11:19089. doi: 10.1038/s41598-021-98267-3 (PMC8476490; doi:10.1038/s41598-021-98267-3)
Supplement: Supplementary file 1 — Supplementary Information 1. [file 41598_2021_98267_MOESM1_ESM.docx]

**Integration and gene co-expression network analysis of scRNA-seq transcriptomes reveal heterogeneity and key functional genes in human spermatogenesis**

Najmeh Salehi^1,2^, Mohammad Hossein Karimi-Jafari^3^, Mehdi Totonchi^1,2*^, Amir Amiri-Yekta^1*^

^1^ Department of Genetics, Reproductive Biomedicine Research Center, Royan Institute for Reproductive Biomedicine, ACECR, Tehran, Iran.

^2^ School of Biological Science, Institute for Research in Fundamental Sciences (IPM), Tehran, Iran.

^3^ Department of Bioinformatics, Institute of Biochemistry and Biophysics, University of Tehran, Tehran, Iran.

^*^ Correspondence: [m.totonchi@royaninstitute.org](mailto:m.totonchi@royaninstitute.org), [amir.amiriyekta@royaninstitute.org](mailto:amir.amiriyekta@royaninstitute.org)


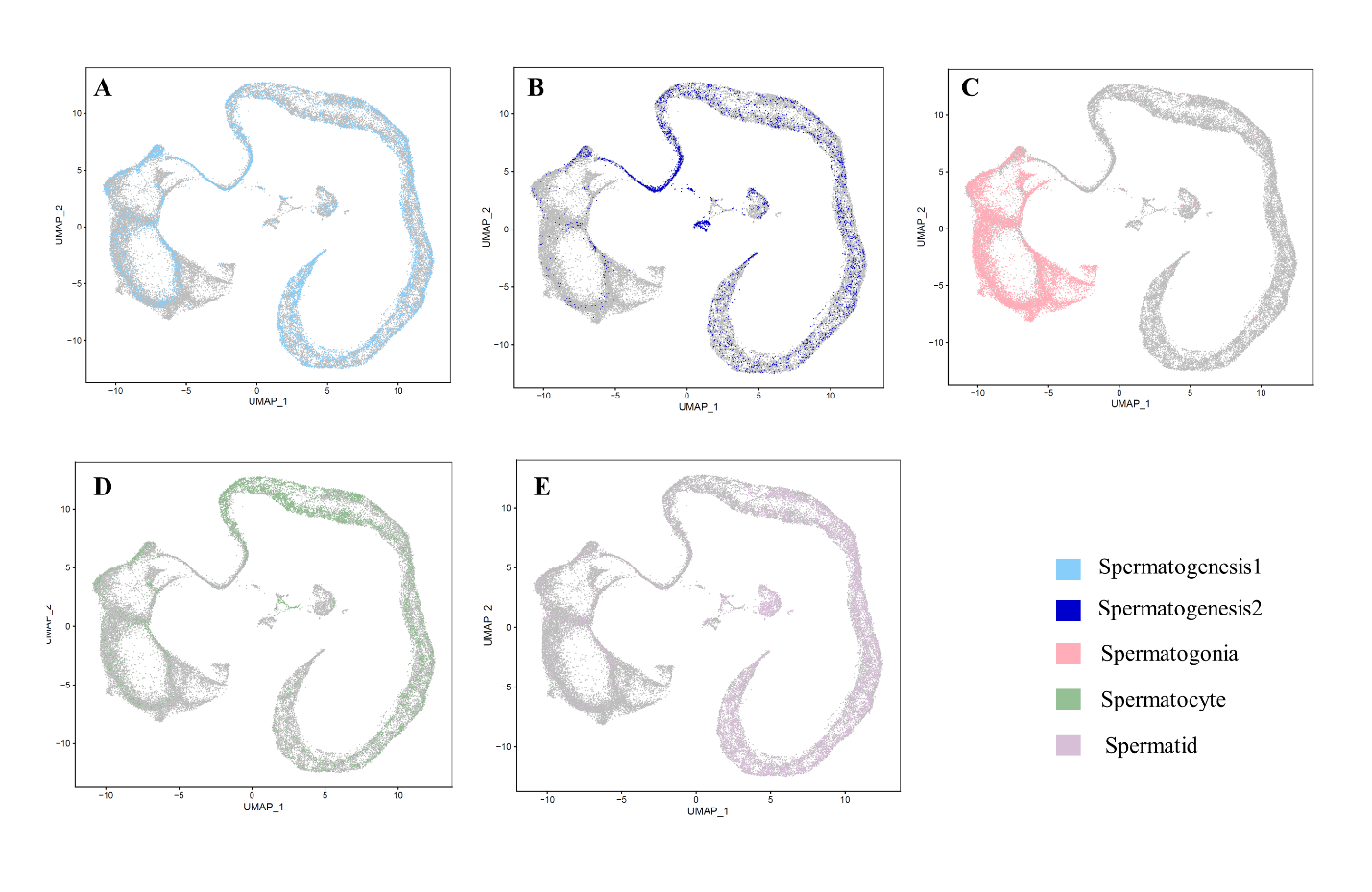


**Figure S1.** The UMAP plots of integrated human testicular cells data, cells were colored based on the five different used datasets in this study from A to E.


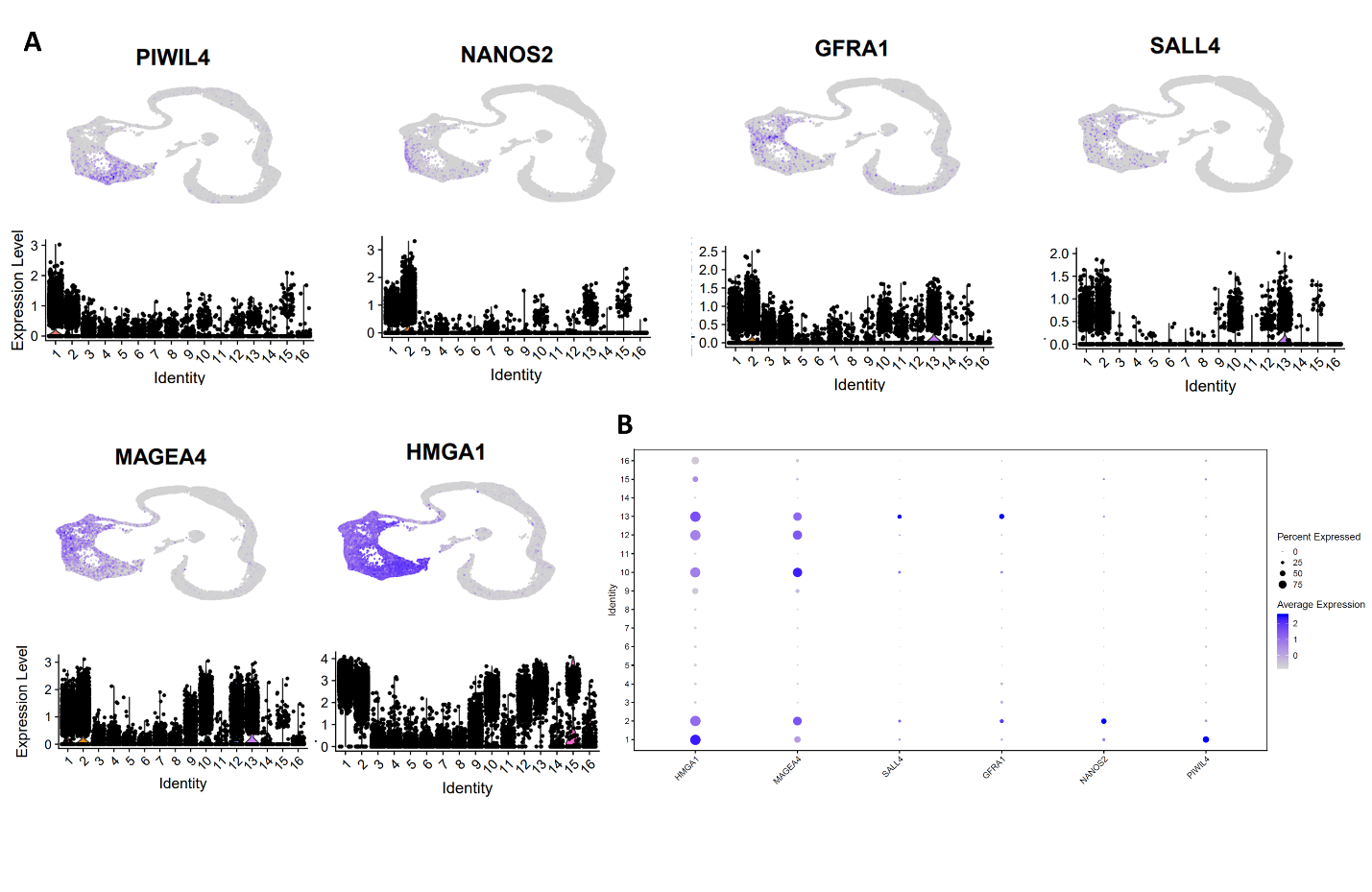


**Figure S2.** Expression of spermatogonia markers. (A) Gene expression patterns of spermatogonia markers in the UMAP space and their expression levels in each of 16 cluster. (B) Dot-plot presentation of markers expression in each of 16 cluster.


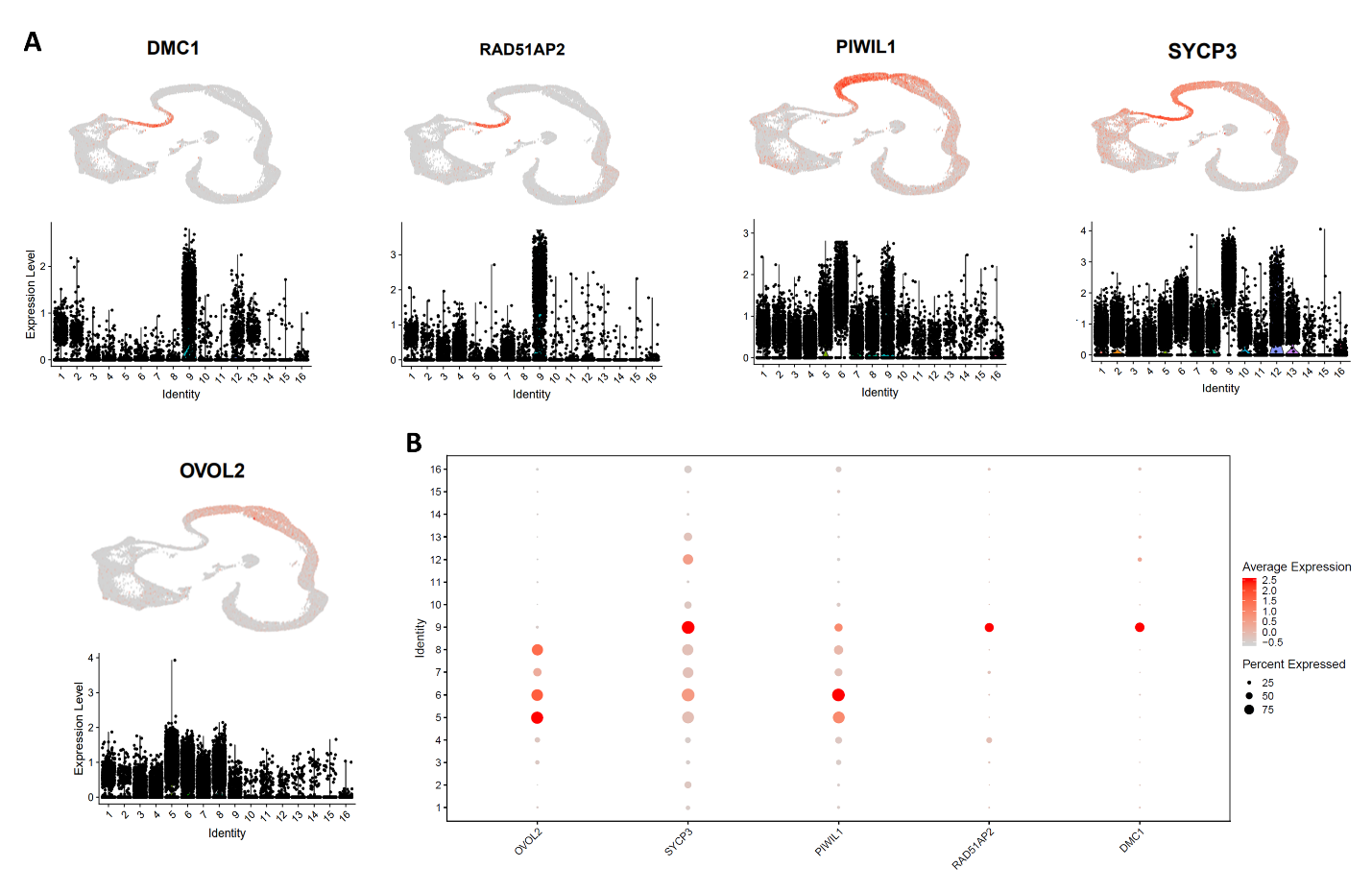


**Figure S3.** Expression of spermatocyte markers. (A) Gene expression patterns of spermatocyte markers in the UMAP space and their expression levels in each of 16 cluster. (B) Dot-plot presentation of markers expression in each of 16 cluster.


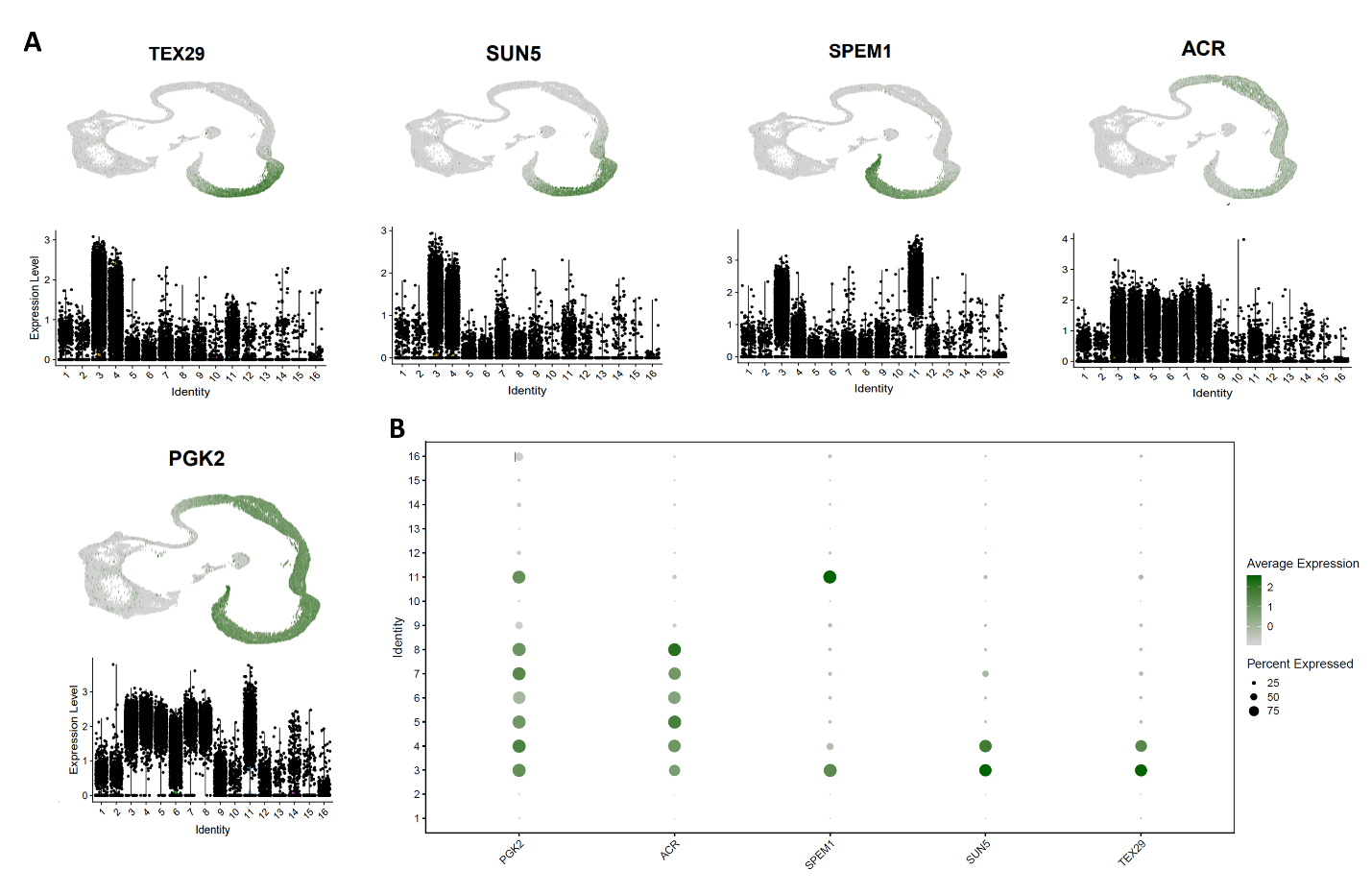


**Figure S4.** Expression of spermatid markers. (A) Gene expression patterns of spermatid markers in the UMAP space and their expression levels in each of 16 cluster. (B) Dot-plot presentation of markers expression in each of 16 cluster.


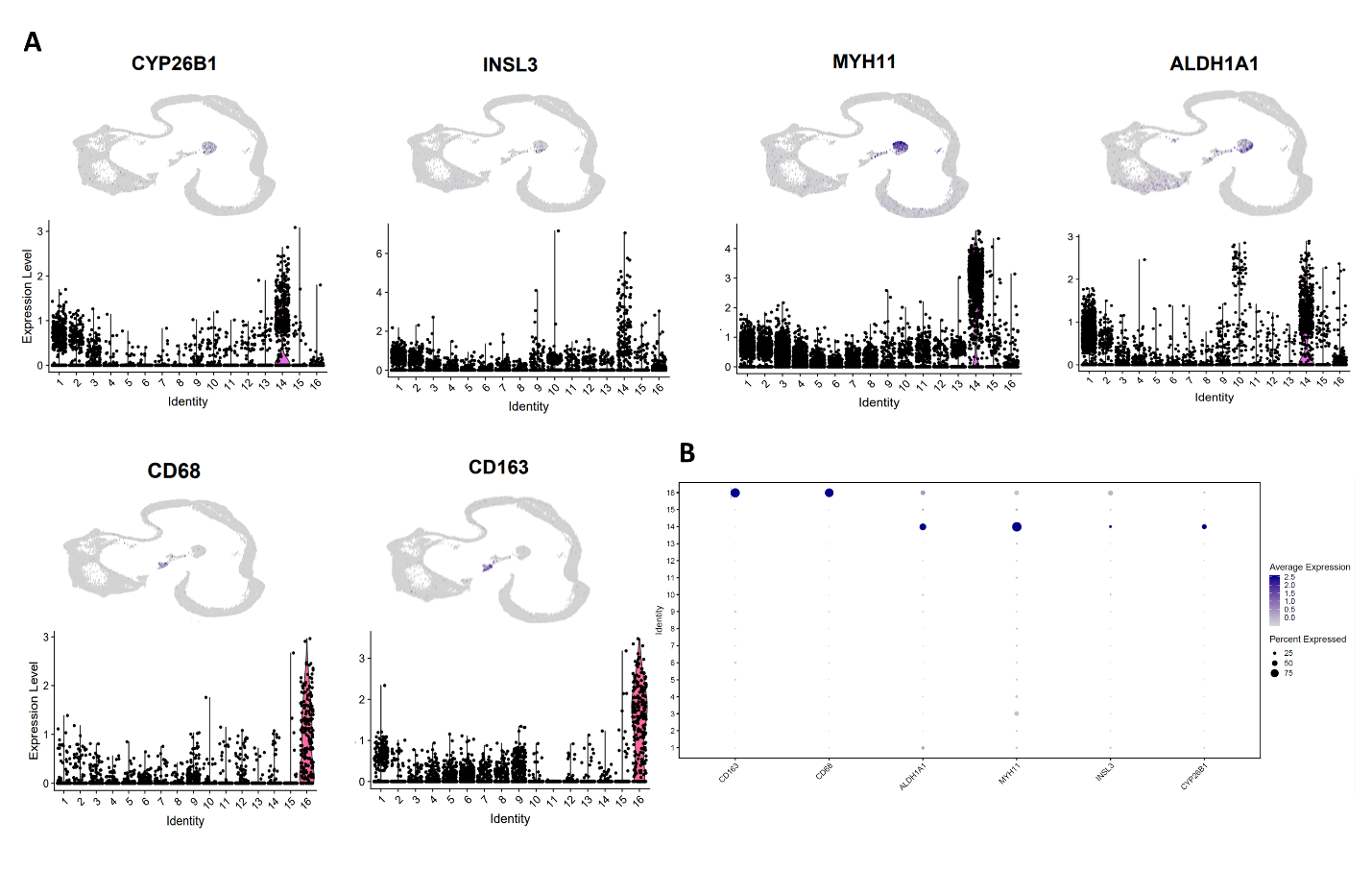


**Figure S5.** Expression of somatic cells markers. (A) Gene expression patterns of somatic cell markers in the UMAP space and their expression levels in each of 16 cluster. (B) Dot-plot presentation of markers expression in each of 16 cluster.


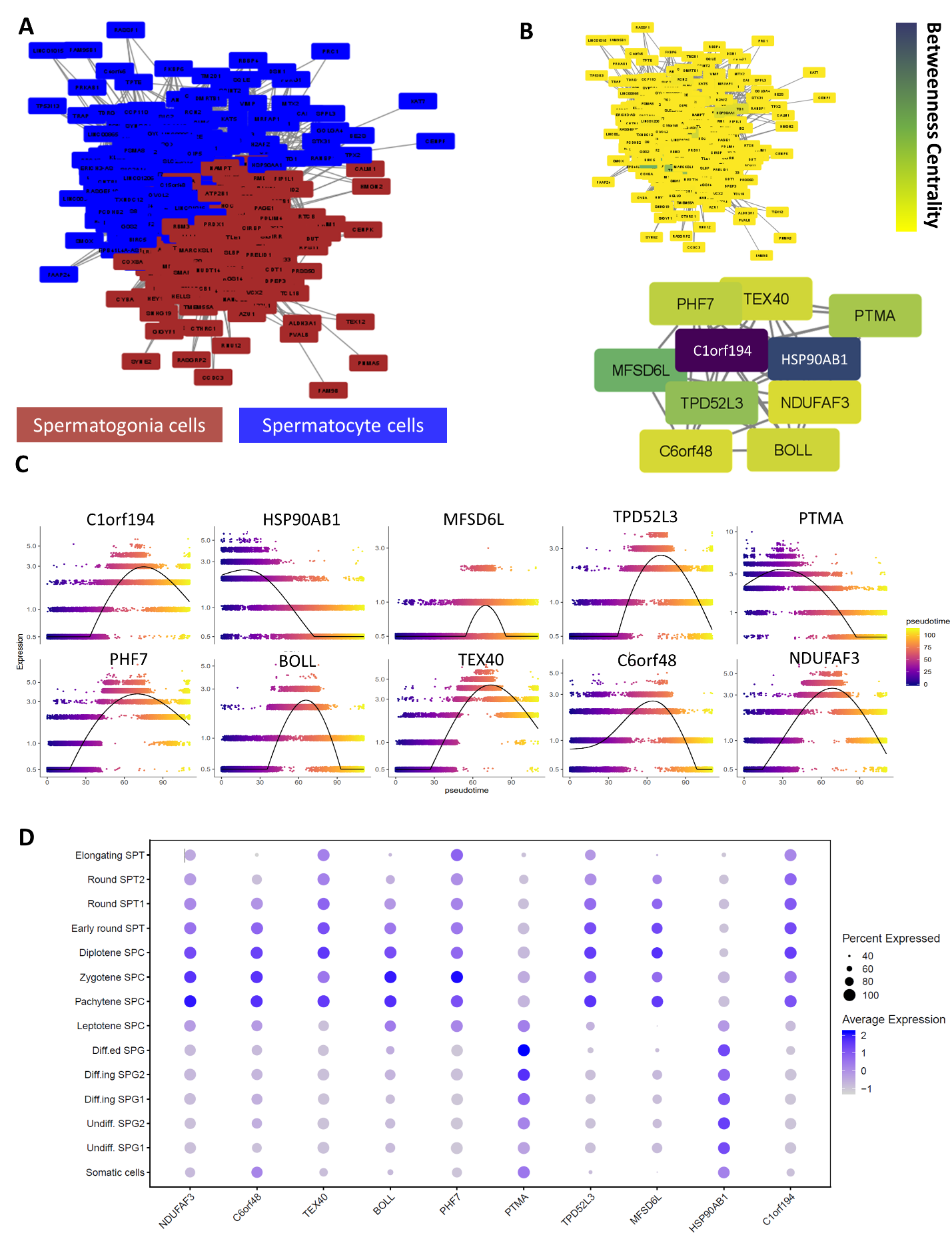


**Figure S6.** Betweenness centrality analysis of spermatogonia and spermatocyte modules in the weighted gene co-expression network (WGCN). (A) The presentation of spermatogonia and spermatocyte modules in the spermatogenesis WGCN. The relation between colored modules and cell types were shown in the inset figure. (B) The spermatogonia and spermatocyte modules are colored based on the BCs from yellow to purple. The top ten genes with the highest BCs between spermatogonia and spermatocyte modules are highlighted. The expressions of the top BCs genes between the spermatogonia and spermatocyte along (C) the pseudotime and (D) the cell-types clusters.


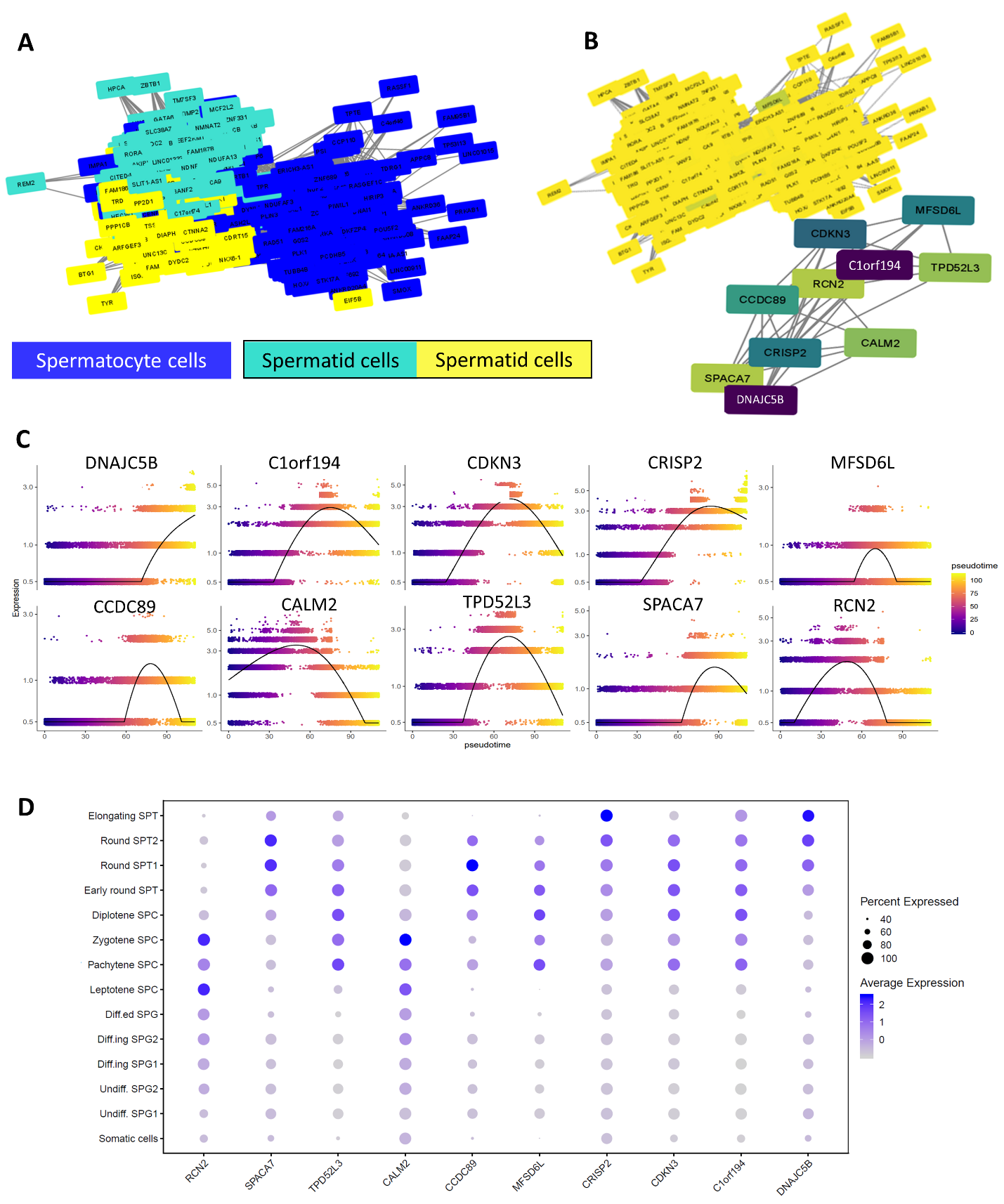


**Figure S7.** Betweenness centrality analysis of spermatocyte and spermatid modules in the weighted gene co-expression network (WGCN). (A) The presentation of spermatocyte and spermatid modules in the spermatogenesis WGCN. The relation between colored modules and cell types were shown in the inset figure. (B) The spermatocyte and spermatid modules are colored based on the BCs from yellow to purple. The top ten genes with the highest BCs between spermatocyte and spermatid modules are highlighted. (C) The expressions of top BCs genes between the spermatocyte and spermatid modules along (C) the pseudotime and (D) the cell-types clusters.
